# Antiplatelet Therapy Combined with Anastrozole Induces Features of Partial EMT in Breast Cancer Cells and Fails to Mitigate Breast-Cancer Induced Hypercoagulation

**DOI:** 10.3390/ijms22084153

**Published:** 2021-04-16

**Authors:** Kutlwano R. Xulu, Tanya N. Augustine

**Affiliations:** School of Anatomical Sciences, Faculty of Health Sciences, University of the Witwatersrand, 7 York Road, Parktown, Johannesburg 2193, South Africa

**Keywords:** breast cancer, hypercoagulation, aspirin, clopidogrel, atopaxar, anastrozole, platelet activation, thrombin activity, tumour survival

## Abstract

Thromboembolic complications are a leading cause of morbidity and mortality in cancer patients. Cancer patients often present with an increased risk for thrombosis including hypercoagulation, so the application of antiplatelet strategies to oncology warrants further investigation. This study investigated the effects of anastrozole and antiplatelet therapy (aspirin/clopidogrel cocktail or atopaxar) treatment on the tumour responses of luminal phenotype breast cancer cells and induced hypercoagulation. Ethical clearance was obtained (M150263). Blood was co-cultured with breast cancer cell lines (MCF7 and T47D) pre-treated with anastrozole and/or antiplatelet drugs for 24 h. Hypercoagulation was indicated by thrombin production and platelet activation (morphological and molecular). Gene expression associated with the epithelial-to-mesenchymal transition (EMT) was assessed in breast cancer cells, and secreted cytokines associated with tumour progression were evaluated. Data were analysed with the PAST3 software. Our findings showed that antiplatelet therapies (aspirin/clopidogrel cocktail and atopaxar) combined with anastrozole failed to prevent hypercoagulation and induced evidence of a partial EMT. Differences in tumour responses that modulate tumour aggression were noted between breast cancer cell lines, and this may be an important consideration in the clinical management of subphenotypes of luminal phenotype breast cancer. Further investigation is needed before this treatment modality (combined hormone and antiplatelet therapy) can be considered for managing tumour associated-thromboembolic disorder.

## 1. Introduction

The relationship between tumour cells and platelets is complex, reciprocal, and implicated in driving hypercoagulation as a factor of tumorigenesis. Cancer patients typically present with thrombocytosis and features associated with hypercoagulation, and they are thus are at risk for thromboembolic complications, a leading cause of morbidity and mortality [1,2]. In post-menopausal patients presenting with hormone-dependent breast cancer, anastrozole (a non-steroidal aromatase inhibitor) is typically a first line treatment; however, while this drug improves survival, it is associated with thrombotic risk [3,4,5]. The addition of antiplatelet therapy to the management of cancer has been suggested; however, there remains a dearth of information on the efficacy of combined antiplatelet and hormone therapy on hypercoagulation and reciprocal effects on cancer.

The pathophysiology of thrombosis is underpinned by the dysregulation of the coagulation cascade and activities of cellular constituents including endothelial cells and platelets. Platelets are anucleate derivatives of megakaryocytes, approximately 2–3 μm in diameter with an average lifespan of seven days within the circulation [6]. Platelets present with a range of bioactive molecules including various cytokines, chemokines, and platelet agonists sequestered within granules, the most numerous of which are α-granules, followed by the electron-dense δ-granules, and, finally, lysosomes [7]. Platelets are able to increase the expression of tissue factor (TF) on tumour cells, therefore enabling tumour cells to activate the coagulation cascade, which, in turn, maintains an activated state in platelets that facilitates thrombosis [8,9,10,11] and ensures a steady supply of growth factors needed for tumour progression through platelet signalling [12,13,14]. TF activates factor X upon exposure to circulating factor VII, an activation cascade that leads to thrombin production [15,16]. Tumour-derived microparticles and exosomes are also a source of various substances, including CD63, platelet-derived growth factor (PDGF), vascular endothelial growth factor (VEGF), and thrombin, that promote tumour-cell induced platelet aggregation (TCIPA) [17,18] and maintain a hypercoagulable state [8,9,10,11]. Ultimately, within the circulation, tumour cell-secreted agonists enable platelet aggregation, protecting tumour cells from shear forces and immunosurveillance [19]. The translocation of adhesion proteins (including GPIb complex, GPIIb/IIIa, and CD62p) from α-granules to the platelet membrane are regarded as the hallmarks of early activation, with the subsequent release of lysosomal membrane proteins (LAMP2/3 and CD63) from δ granules occurring at a later stage of activation [7,20]. This is typically coupled with the direct or indirect (microparticle) release of agonists to further enhance the platelet activation and aggregation process. This includes the secretion of growth factors involved in inflammation and wound healing that are then subverted for tumour progression [21,22].

Antiplatelet strategies aimed at reducing platelet activation and aggregation have shown considerable benefit in reducing thromboembolic complications in cardiovascular disease [23], so the application of such strategies to oncology, while currently not standard protocol, bears consideration. The antiplatelet therapies of aspirin, clopidogrel, and atopaxar used in this study target the following three main pathways, respectively, involved in platelet activation: (a) the activation of cyclooxygenase 1 (COX-1), which facilitates thromboxane (TXA2) synthesis; (b) the engagement of the P2Y12 receptors by adenosine diphosphate (ADP); and (c) the activation of protease-activated receptor 1 (PAR1) by thrombin [24,25,26]. The impact of such drugs in breast cancer with or without cancer therapy remains uncertain. Breast cancer cells express targets for these antiplatelet therapies: COX enzymes [27,28], P2Y12 ADP-receptors [29], and PAR1 receptors [30]. It is noteworthy that these antiplatelet drug targets also mediate tumour functions such as oestrogen-driven cell proliferation through COX-2 [27,28,31], inflammation and immune modulation through P2Y12 receptors [32,33], and the epithelial–mesenchymal transition (EMT) through the overexpression of PAR1 receptors [34]. Thus, a combined hormone and antiplatelet therapy would have reciprocal effects on hypercoagulation and breast cancer outcome. MCF7 and T47D cell lines, which have been shown to express P450 enzymes, are capable of metabolising various drugs [35]. These cell lines are thus a suitable model in which to the investigate antiplatelet therapy effects on the tumour cell-mediation of thrombotic complications and tumour progression itself.

Aspirin has been noted to have a moderate impact in the reduction of the metastasis of mammary carcinomas [36,37], while aspirin combined with tamoxifen has been shown to provide cardio-protective effects and may reduce breast cancer risk [38]. Clopidogrel has conversely been associated with the risk of development of neoplasms in patients presenting with cardiovascular disease [39]. In addition, the SPS3 and DAPT clinical trials showed that the prolonged use of clopidogrel increased cancer-related deaths [40]. Moreover, the dual administration of aspirin and clopidogrel, which is highly recommended in cardiovascular disease [24], was shown to heighten breast tumour aggressiveness in an animal model through increased vascular mimicry associated with high mortality rates [41]. In contrast, the CHARISMA clinical trial showed that the combination of aspirin and clopidogrel decreased cancer incidence and cancer-related mortality [42]. However, clopidogrel combined with tamoxifen (hormone therapy) and chemotherapy agents (cisplatin and doxorubicin) increased metastasis to the lung in a mouse model [43]. Little is known regarding the role of atopaxar in tumour progression; however, it has been shown to inhibit the Janus kinase-signalling pathway (JAK–STAT), which promotes cell division and neoplastic transformation in cells, thus suggesting a potential role for atopaxar in reducing tumour formation [44]. Despite this, its impact when combined with hormone therapy is not yet known and warrants further investigation. We thus present findings demonstrating the effects of combined antiplatelet therapy and hormone therapy, specifically anastrozole, on hypercoagulation and tumour profiles.

## 2. Results

### 2.1. Anastrozole Combined with Aspirin and Clopidogrel (AnaAspClop) Promotes a Hypercoagulable State

**Figure 1:**
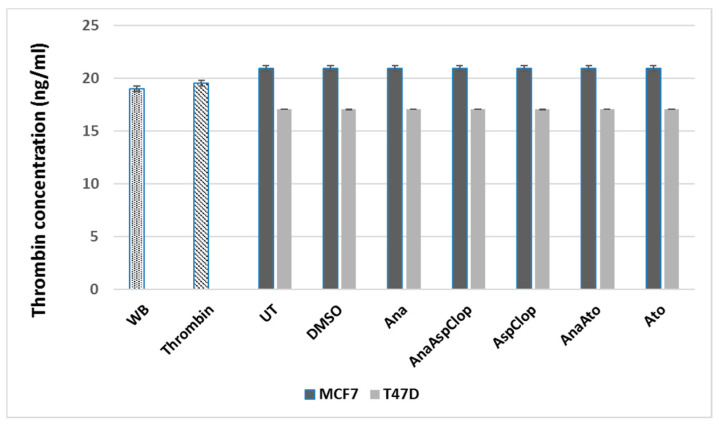
Thrombin generation (ng/mL) in whole blood exposed to combined anastrozole (Ana) and antiplatelet therapy (AspClop (aspirin and clopidogrel) and Ato (atopaxar)) with corresponding controls (baseline = WB; positive = exogenous thrombin; media = untreated; diluent = DMSO; hormone = Ana; antiplatelet = AspClop/Ato). Thrombin generation was higher in whole blood exposed to MCF7 cells than T47D cells, despite the treatment used.

Thrombin is a known agonist of platelet activation, acting by binding to PAR receptors on platelets and facilitating fibrin formation from fibrinogen [45,46]. Baseline levels of thrombin were detected in pure whole blood (Figure 1). Thrombin generation was induced in whole blood exposed to breast cancer cells (MCF7 and T47D), thus promoting a hypercoagulable state. No significant differences were detected in levels of thrombin generated among the treatment groups (anastrozole, aspirin, and clopidogrel (AnaAspClop)) and controls; however, thrombin levels were generally higher in whole blood exposed to MCF7 cells than in whole blood exposed to T47D cells (Figure 1). This finding suggests that differences in thrombin levels were dependent on the type of cell line rather than the treatment and highlights differences in the capacity of MCF7 and T47D cells to induce a hypercoagulable state.

**Figure 2:**
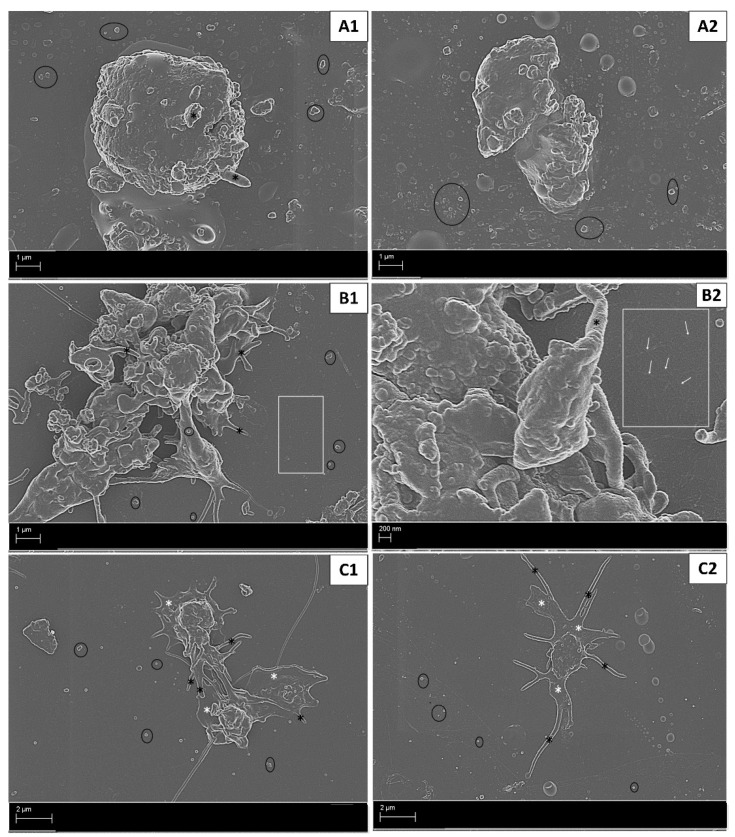
Platelet activation in controls, (**A1**,**2**) untreated whole blood showing levels of baseline platelet activation, (**B1,2**) whole blood exposed to 0.1 U/mL thrombin (positive control) showing advanced platelet activation with clotting and fibrin formation, and (**C1**,**2**) whole blood exposed to DMSO-treated MCF7 cells (**C1**) and T47D cells (**C2**) showed early stages of platelet activation shown by the pseudopodia formation of varying sizes. Annotations: black * = pseudopodia; black circle = microparticles; white rectangle (with/without white arrows) = fibrin formation; white * = hyalomere spread.

**Figure 3:**
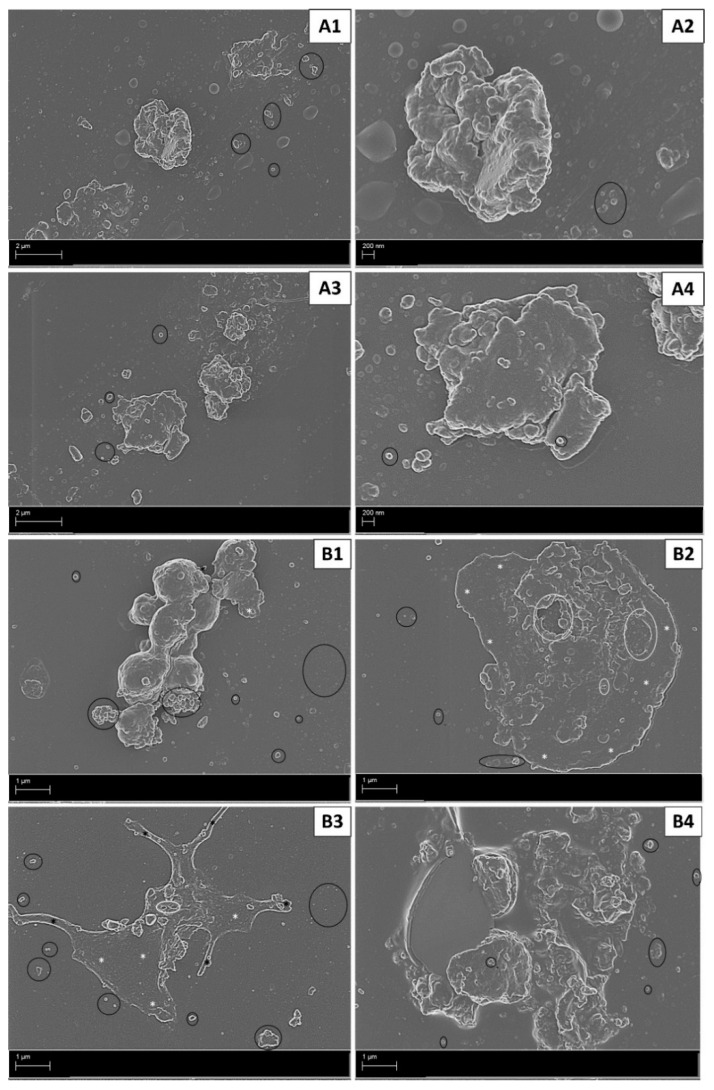
Platelet activation in media control (**A1**–**4**) and hormone control (anastrozole = Ana) (**B1**–**4**). Early stages of platelet activation shown by irregular platelets and some microparticle release induced in platelets exposed to MCF7 cells (**A1**,**2**) and T47D cells (**A3**,**4**) in media. Whole blood exposed to anastrozole-treated MCF7 cells (**B1**,**2**) and T47D cells (**B3**,**4**) induced advanced stages of platelet activation, as indicated by platelet aggregates (**B1,4**), fully spread hyalomeres with distinct open canaliculi systems (**B2**,**3**), and fewer pseudopodia (**B3**). Annotations: black * = pseudopodia; black circle = microparticles; white * = hyalomere spread.

In untreated whole blood, an ultrastructural analysis revealed baseline levels of platelet activation as indicated by a rounded morphology, with short pseudopodia and few microparticles (Figure 2A1,2), although some platelet clumping was noted (Figure 2A2). In contrast, whole blood exposed to exogenous thrombin (positive control) revealed advanced stages of platelet activation indicated by clot formation, formed by platelets forming clumps (Figure 2B1) and evidence of fibrin formation in the extracellular milieu (Figure 2B2). The diluent control (DMSO) induced platelet pseudopodia formation with some hyalomere formation and microparticle release (Figure 2C1,2). The media control in both cell lines (MCF7 and T47D) induced an irregular platelet morphology with microparticle formation, suggesting early stages of activation (Figure 3A1–4).

**Figure 4:**
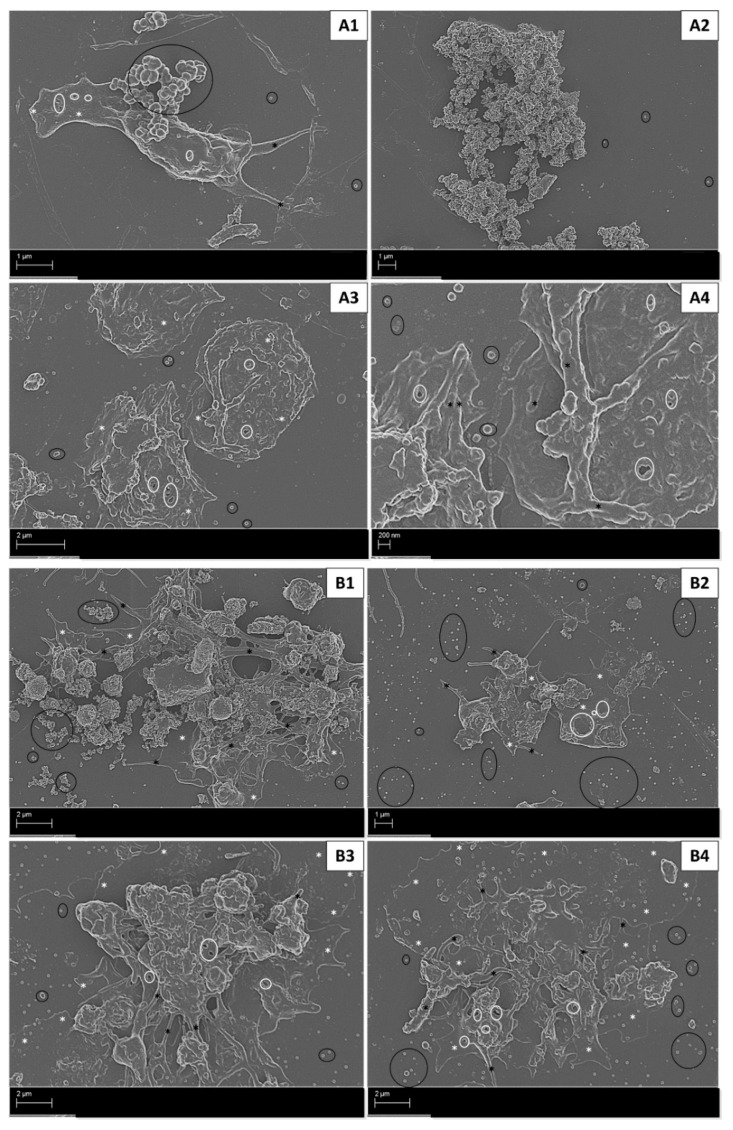
Platelet activation in whole blood exposed to AnaAspClop-treated MCF7 cells (**A1**,**2**) and T47D cells (**A3**,**4**), as well as corresponding antiplatelet control (AspClop), (**B1**,**2**) (MCF7 cells) and (**B3**,**4**) (T47D cells). Advanced stages of platelet activation were induced with the AnaAspClop treatment in both cell lines; however, less platelet clumping/clot formation was evident compared to the antiplatelet control. Annotations: black * = pseudopodia; black circle = microparticles; white rectangle (with/without white arrows) = fibrin formation; white * = hyalomere spread.

In the AnaAspClop treatment group, MCF7 cells induced activated platelets with hyalomere spread and pseudopodia formation. This was associated with prominent open canaliculi systems (OCSs) and large microparticle release (Figure 4A1). Large clumps of round small structures (reminiscent of globular fibrin or microparticles) that varied in size were also evident (Figure 4A2). This treatment caused less advanced stages of platelet activation compared to the hormone (anastrozole) control (Figure 3B1,2) and antiplatelet control (AspClop), which showed clumped platelets as evidence of clot formation, and platelets with fully spread hyalomeres, which indicated more advanced stages of activation (Figure 4B1,2). The T47D cells under the AnaAspClop treatment induced more fully spread hyalomeres in platelets with prominent OCSs and more microparticle secretion (Figure 4A3,4); however, in hormone (anastrozole) (Figure 3B3,4) and antiplatelet controls (AspClop) (Figure 4B3,4), more clumped platelets were evident. Hypercoagulation and the variation in platelet activation, as demonstrated by ultrastructural analysis, were further illustrated by an assessment of platelet activation markers and the index of platelet activation (IPA).

The total CD62p IPA (Table 1) was found to be higher in MCF7 cells compared to T47D cells (albeit not significantly), corresponding with the ultrastructural changes observed in platelets (Figure 4). The hyalomere spread in platelets exposed to T47D-treated cells reduced the levels of detectable CD62p by flow cytometry, as this molecule in known to facilitate adhesion and spreading [47,48]. The total CD63 IPA was also found to be higher in platelets exposed to MCF7 cells compared to T47D cells, albeit not significantly. CD63 is known to mediate early and final events of platelet activation such as platelet recruitment and fibrinolysis [6,49]. Furthermore, CD63 is contained within various sources, including microparticles [3,30]. The high levels of the CD63 IPA in the whole blood exposed to MCF7 cells corresponded with the higher levels of observed microparticles (Figure 3A1,2). These results prompted the investigation of the relationship between the CD62p and CD63 IPA. While the total IPA represented levels of CD62p and CD63 expression, in its totality within quadrant 3 (Figure 8), an assessment of the IPA within intervals indicated the differential expression of these markers among platelets.

Under treatment with AnaAspClop for both cell lines (MCF7 and T47D), the CD62p IPA within intervals 1 and 2 was generally higher compared to the antiplatelet therapy control (AspClop) but lower compared to the hormone therapy control (Ana) and the media control within interval 2 (Table 1). However, the AnaAspClop treatment of MCF7 cells reduced the CD62p IPA in interval 3 in relation to other treatments, and this reduction was significant when compared to the antiplatelet therapy control (AspClop) and the media control (Table 1). In contrast, in T47D cells, the induced CD62p IPA under AnaAspClop treatment remained high in relation to the antiplatelet therapy (AspClop) and the hormone therapy control (Ana), albeit not significantly. In interval 4, AnaAspClop treatment in MCF7 cells induced a higher CD62p IPA compared to the hormone control (Ana, *p* > 0.05) and T47D cells (*p* < 0.05) but a lower IPA than in the antiplatelet therapy control (AspClop) (Table 1). Despite some differences, both cell lines induced a similar trend in the CD62p IPA with the AnaAspClop treatment, which showed a peak in the CD62p IPA within interval 2 followed by a gradual drop between intervals 3 and 4 (Table 1). AnaAspClop treatment in both cell lines induced a higher CD63 IPA compared to the antiplatelet therapy control (AspClop) in all intervals (1–4), except for T47D cells in interval 3, which reduced the CD63 IPA in relation to the antiplatelet therapy control (AspClop) (Table 2). When compared to the hormone therapy control (Ana), the AnaAspClop treatment in MCF7 cells induced a higher CD63 IPA within intervals 1 and 3 and reduced the CD63 IPA within intervals 2 and 4 (Table 2). In contrast, AnaAspClop treatment in T47D cells induced a higher CD63 IPA in relation to the hormone therapy control in interval 3 only, and no change was seen in other intervals (Table 2).

At intervals (1 and 2) (data not shown), there was no correlation between the CD62p and CD63 IPA. A correlation analysis revealed that under the combined AnaAspClop treatment, the identified relationship between CD62p and CD63 became weaker, particularly in the T47D group compared to the hormone control; however, it was stronger when compared to antiplatelet control in MCF7 cells (Table 3). This suggests that the cell lines and the combination of anti-platelet therapy with hormone therapy alters platelet response.

### 2.2. Cytokine Secretion with Anastrozole, Aspirin, and Clopidogrel Cocktail

**Figure 5:**
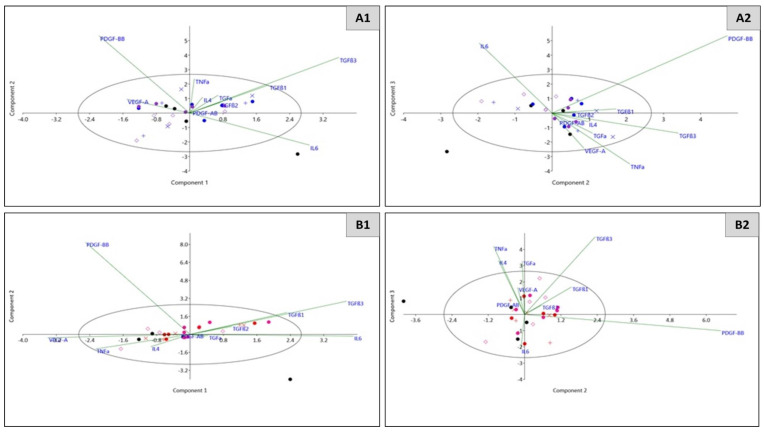
Scatterplot representing the relationship between cytokines (IL1β, TNF-α, PDGF-AB, IL6, PDGF-BB, VEGF-A, IL4, IL10, TGF-α TGFβ -1, -2, -3) following whole blood exposure to AnaAspClop-treated cells (MCF7/T47D) across principal components 1, 2 and 3, on an eigenvalue scale with 95% ellipse. MCF7 cell culture group (A1-2): WB control–black circle, Media control–blue +, Diluent control–blue x, Anas control–blue circle, AspClop control–purple circle, AnasAspClop (EXP)–purple diamond. T47D cell culture group (B1-2): WB control–black circle, Media control–red +, Diluent control–red x, Anas control–red circle, AspClop control–pink circle, AnasAspClop (EXP)–pink diamond.

Principal component analysis (PCA) was used to investigate the relationships between multiple cytokines, as secreted under combined hormone and antiplatelet treatment, within each cell line group. By reducing the variables to three principal components (PCs), we identified which cytokines were responsible for explaining the majority of the variation induced by each cell line (Table 4) and for separating or delineating the treatment groups (Figure 5). For MCF-7 cells, IL-6 and TGF-β3 gave positive contributions to PC1 (Table 4), with the latter appearing primarily responsible for discriminating the control groups from the experimental group (AnaAspClop) (Figure 5). This concept was further illustrated by PC2, in which PDGF-BB and TGF-β3 were positively loaded, and PC3, in which PDGF-BB and IL-6 had strong positive contributions in separating out the experimental group from controls (Table 4 and Figure 5). For T47D cells, in addition to IL-6 and TGF-β3, which gave strong positive contributions to PC1 (Table 4) and appeared primarily responsible for clustering of the AspClop and Ana controls, this was affected by the negative loading of VEGF-A, which also separated out the experimental group (AnaAspClop). Similar to the loadings seen for MCF-7 cells in PC2, T47D cells showed a strong positive contribution from PDGF-BB, which was associated with a right shift, thus distinguishing the experimental group. PC3 had positive contributions from a number of cytokines including IL-4, TNF-α, TGF-α, and TGF-β3; however, their discriminatory power was unclear, echoing the total explained variance of 72.79%.

### 2.3. Anastrozole- and Atopaxar-Mediated Effects on Hypercoagulation

**Figure 6:**
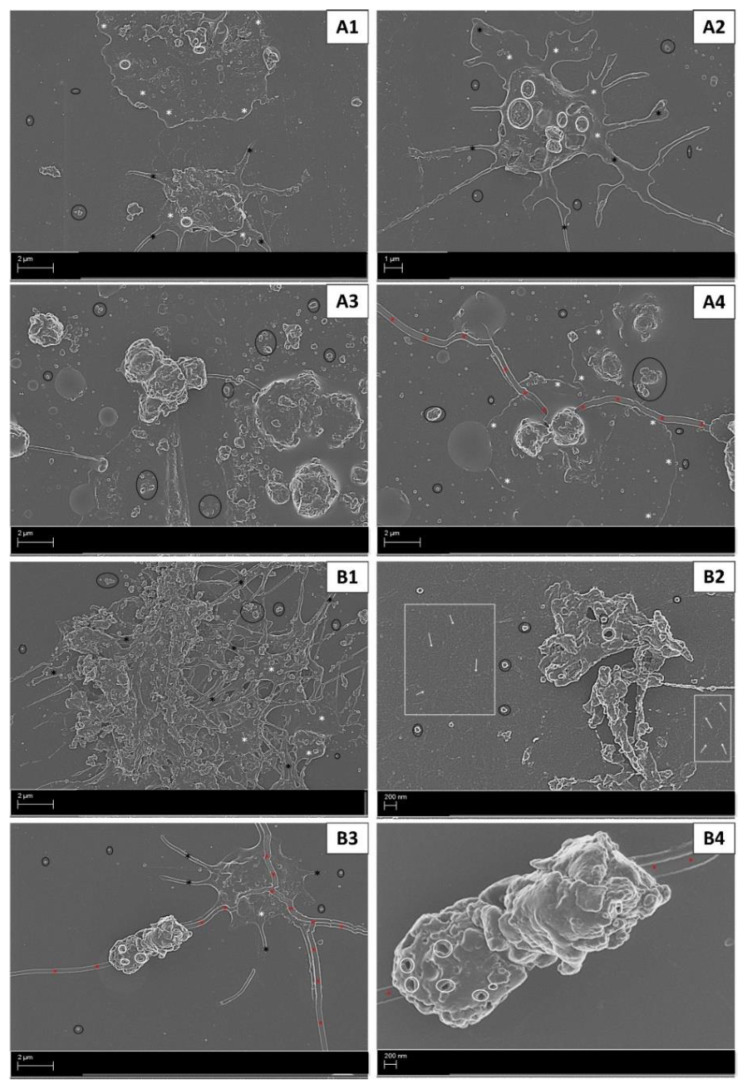
Platelet activation in whole blood exposed to AnaAto-treated MCF7 cells (**A1**,**2**) and T47D cells (**A3**,**4**), as well as corresponding antiplatelet control (Ato) (**B1**,**2**) MCF7 cells and (**B3**,**4**) T47D cells. AnaAto-treated MCF7 cells induced fully spread hyalomeres in platelets, whereas T47D cells induced platelet aggregates. The antiplatelet control (Ato) in MCF7 cells induced more advanced stages of activation (clot formation), whereas in T47D cells, similar ultrastructural changes compared to the AnaAto treatment were induced. Annotations: black * = pseudopodia; black circle = microparticles; white rectangle (with/without white arrows) = fibrin formation; white * = hyalomere spread; red * = artefact (cracked glass slide).

AnaAto-treated MCF7 cells induced a mixed platelet morphology characterised by rounded platelets with pseudopodia extensions (early activation) and platelets with fully spread hyalomeres (late stages of activation) (Figure 6A1,2). Furthermore, platelets displayed prominent OCSs, and microparticles were also detected (Figure 6A1,2). In contrast to the hormone (Ana) (Figure 2B1,2) and antiplatelet controls (Ato) (Figure 6B1,2), clumped platelets were not evident. The antiplatelet control, however, also displayed evidence of fibrin formation in the extracellular milieu, indicating hypercoagulation (Figure 6B2). In the AnaAto-treated T47D cells, platelet clumping was more predominant, suggesting clot formation, fully spread hyalomeres, and (consequently) advanced stages of platelet activation (Figure 6A3,4). In contrast, the hormone (Figure 2B3,4) and antiplatelet therapy control (Ato) (Figure 6B3,4) displayed a mixed morphology with platelets at early stages of activation and platelet aggregates.

Investigating corresponding platelet activation markers revealed that under the AnaAto treatment, the total IPA values for CD62p (Table 1) and CD63 (Table 2) were higher in platelets exposed to treated MCF7 cells than T47D cells, albeit not significantly. Within interval 1, the CD62p IPA in AnaAto-treated cells (MCF7 and T47D) was higher than that induced by the antiplatelet control (Ato) but lower than that induced by the hormone control (Ana) (Table 1). In interval 2, the higher levels of the CD62p IPA was maintained in AnaAto-treated MCF7 cells but reduced in the AnaAto-treated T47D cells compared to antiplatelet therapy control (Ato) (Table 1). In contrast, the CD62p IPA in both cell lines was higher compared to that of the hormone control (Ana) (Table 1). In intervals 3 and 4, the CD62p IPA in platelets exposed to AnaAto-treated cells (MCF7 and T47D) was reduced in relation to the antiplatelet control (Ato) (*p* < 0.05 in interval 3) and the hormone control (Ana) (*p* < 0.05 in T47D cells in interval 4) (Table 1). In comparison to the media control, AnaAto resulted in a higher CD62p IPA induced by MCF7 cells and a lower CD62p IPA induced by T47D cells in interval 3 (Table 1). However, in comparison to the antiplatelet therapy control, AnaAto induced a lower CD62p IPA (Table 1). The CD63 IPA in AnaAto-treated MCF7 cells was lower than the antiplatelet therapy control in intervals 1 and 3 but higher in intervals 2 and 4 (Table 2). In comparison to the hormone therapy control in MCF7 cells, the CD63 IPA was lower in intervals 1–3, but higher in interval 4, thus illustrating the highly active platelets and echoing ultrastructural findings (Table 2 and Figure 6). In AnaAto-treated T47D cells, the CD63 IPA was higher than in the antiplatelet therapy control in interval 1 but lower in subsequent intervals (2–4) (Table 2). In comparison to the hormone control, the CD63 IPA was lower in intervals 1 and 4 but higher in intervals 2 and 3 (Table 2). The general trend in the CD63 IPA within intervals following platelet exposure to AnaAto-treated cells showed a gradual increase and peak in the CD63 IPA within intervals 2 and 3, followed by a drop in interval 4 (Table 2).

A correlation analysis revealed that under combined AnaAto treatment, the identified relationship between CD62p and CD63 became weaker in the T47D group in interval 3 compared to the hormone control, but it strengthened in interval 4 where both cell lines evidenced strong correlation (Table 3). This suggests that the cell lines and the combination of anti-platelet therapy with hormone therapy alters platelet response dependent on the cell phenotype and the employed antiplatelet therapy.

### 2.4. Cytokine Secretion with Anastrozole and Atopaxar Treatment

**Figure 7:**
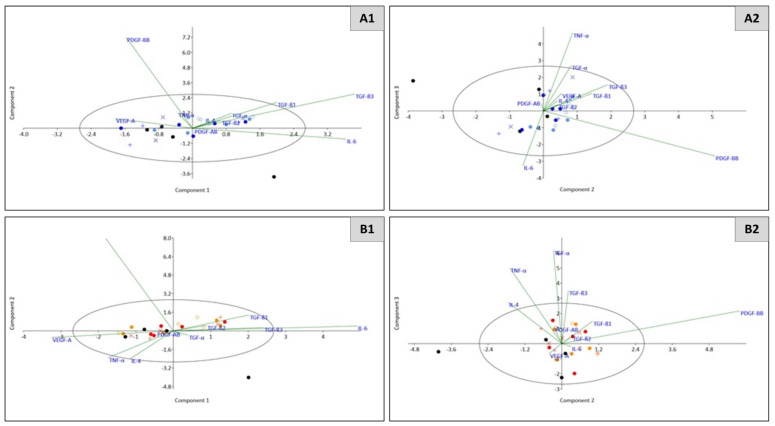
Scatterplot representing the relationship between cytokines (IL1β, TNF-α, PDGF-AB, IL6, PDGF-BB, VEGF-A, IL4, IL10, TGF-α TGFβ -1, -2, -3) following whole blood exposure to AnaAto-treated cells (MCF7/T47D) across principal components 1, 2 and 3, on an eigenvalue scale with 95% ellipse. MCF7 cell culture group (A1-2): WB control–black circle, Media control–blue +, Diluent control–blue x, Anas control–blue circle, AspClop control–purple circle, AnasAspClop (EXP)–purple diamond. T47D cell culture group (B1-2): WB control–black circle, Media control–red +, Diluent control–red x, Anas control–red circle, AspClop control–pink circle, AnasAspClop (EXP)–pink diamond.

For MC7 cells, similar to the previous antiplatelet therapy, IL-6 and TGF-β3 positively contributed to PC1, which was primarily responsible for the right shift in discriminating the experimental group (AnaAto) from the controls (Table 5 and Figure 7). The strong positive contribution from PDGF-BB to PC2, and its negative loading along with IL-6 in PC3 appeared to be responsible for discriminating the media control and diluent controls from those treated with either anastrozole or atopaxar (Table 5). In PC3, a weak positive contribution from TGF-α and a stronger positive contribution from TNF-α were associated with the clustering of the anastrozole control group but otherwise had poor discriminatory value (Table 5 and Figure 7). For T47D cells, similar to the previous antiplatelet therapy, IL-6 had a strong positive contribution to PC1, which led to the clustering of the experimental (AnaAto) group (Table 5). This was also influenced by the negative contribution of VEGF-A to PC1 (Table 5). PC2 showed no significant contributions from cytokines above the cut-off level, although PC3 was affected by positive contributions from TNF-α, TGF-α, and TGF-β3, which (though low in discriminatory power) highlighted the inflammatory microenvironment.

### 2.5. Effects of Reciprocal Interactions between Whole Blood and Breast Cancer Cells on Tumour Progression

#### 2.5.1. Anastrozole-, Aspirin-, and Clopidogrel-Mediated Effects

The effect of combined hormone and antiplatelet therapy on the expression of EMT markers in breast cancer cells exposed to whole blood was assessed. The AnaAspClop treatment downregulated the expression of TGFβ1 in both cell lines (Table 6); in T47D cells, this was significant compared to baseline (media control—cells not exposed to whole blood) and cells exposed to whole blood only (Table 6). It is noteworthy that the downregulation of TGFβ1 with the AnaAspClop treatment was not associated with the loss of the TGFβ1 protein, as shown by the cytokine analysis, because these levels were unaffected (Table 4). With the AnaAspClop treatment, E-cadherin was upregulated in MCF7 cells but downregulated in T47D cells (significant when compared to the diluent) (Table 6). In contrast, the mesenchymal markers N-cadherin and vimentin were upregulated in both cell lines, indicating that the treatment and exposure to whole blood induced an EMT. N-cadherin levels were significantly higher in MCF7 cells when compared to the diluent, whereas vimentin levels were significantly higher in T47D cells compared to the antiplatelet control (AspClop) (Table 6). These findings suggested that this treatment may promote the acquisition of mesenchymal features in MCF7 cells without changing the epithelial phenotype (partial EMT). The induced cytokine secretion with this treatment (AnaAspClop) in MCF7 cells showed that IL6, PDGF-BB, and TGFβ3 facilitated this transformation (Table 4). Moreover, while TGFβ1 was not a major player, that its levels were not significantly affected may explain the maintenance of epithelial features. In contrast, the AnaAspClop treatment may have induced the acquisition of a mesenchymal phenotype in T47D cells. This corresponded with the cytokine secretion induced by this cell line, which showed the following cytokines as playing an important role in mediating induced effects with this treatment: IL6, IL4, TNFα, VEGF-A, PDGF-BB, TGFα, and TGFβ3 (Table 4).

#### 2.5.2. Anastrozole- and Atopaxar-Mediated Effects

AnaAto treatment downregulated TGFβ1 expression in both cell lines (*p* > 0.05) (significant when compared to diluent in T47D cells only) (Table 6), although the protein expression of TGFβ1, as found in cytokine analysis, was evident. E-cadherin was significantly upregulated in MCF7 cells; in contrast, it was significantly downregulated in T47D cells at the matched treatment, baseline, and diluent control (Table 6). An assessment of mesenchymal markers revealed that N-cadherin was also significantly upregulated in MCF7 cells compared to T47D cells, and it was similarly downregulated in T47D cells compared to the baseline (Table 6). Following this pattern, vimentin levels were upregulated in MCF7 cells (*p* > 0.05) (Table 6) and significantly downregulated in T47D cells compared to the baseline and diluent control (Table 6). Taken together, these findings suggest that AnaAto treatment may promote the mesenchymal phenotype in MCF7 cells while maintaining epithelial differentiation (partial EMT). This corresponded with the cytokine secretion profile, which showed that IL6-, TNFα-, PDGF-BB-, TGFα-, and TGFβ3-mediated responses with this treatment (Table 5). In contrast, since it reduced both epithelial and mesenchymal-associated genes in T47D cells, this suggests that this treatment may slow down the rate of phenotypic changes and potentially promote tumour progression, as suggested by the cytokine secretion that showed that the TNFα, VEGF-A, TGFβ3, and TGFα cytokines (Table 5) play important roles in mediating AnaAto-induced responses in T47D cells.

## 3. Discussion

The relationship between breast cancer and thrombosis is well-established [50,51,52,53,54]. Anastrozole is commonly used as a hormone therapy agent in post-menopausal women with breast cancer, but, despite increasing survival, it is also associated with an increased risk of thrombosis [21]. Antiplatelet therapy, while an effective treatment for thromboembolic disorders associated with cardiovascular disease, does not form part of standard therapy in cancer [38,55,56]. Moreover, the effect of antiplatelet therapy using an aspirin and clopidogrel cocktail and atopaxar in combination with hormone therapy in cancer-associated thromboembolic disorders and parameters of tumour progression is not well-known. This study thus assessed the effects of these combined treatments on breast cancer cells exposed to whole blood in order to mimic the interaction of tumour cells with platelets in situ [50]. The findings of this study showed that combined anastrozole and antiplatelet therapy did not prevent hypercoagulation or mitigate against cancer survival, highlighting the complex and reciprocal interactions between breast cancer and vascular components.

In this study, an aspirin and clopidogrel cocktail combined with anastrozole failed to prevent hypercoagulation and induced higher thrombin levels detectable in whole blood than anastrozole-treated cells, with MCF7 cells generally inducing higher thrombin levels. In contrast, thrombin levels were reduced compared to the antiplatelet therapy control (aspirin and clopidogrel), which may have been indicative of heightened thrombin binding [57,58,59] that reflected a more hypercoagulable state. This finding was corroborated by higher levels of clot formation, further showing that, similar to hormone therapy, antiplatelet therapy alone may not be sufficient to reduce the risk of hypercoagulation, thereby revealing a greater role for breast cancer cells in driving thromboembolic complications [60,61,62]. While thrombin is known to be the most potent agonist of platelet activation [63,64] and thus may be responsible for promoting the coagulation process via platelet PAR receptors [64], there are additional avenues by which platelets can be activated [65].

Platelet activation studies have reported heterogeneity in platelet response due to their involvement in multiple functions such as haemostasis, coagulation, inflammation, and angiogenesis [66]. Platelets can also display variability in activation status, including presenting a series of reversible events [63,64], which may be reflected morphologically. In this study, evidence of platelet heterogeneity was displayed in the differential expression of the platelet activation markers CD62p and CD63 (generally lower levels of CD62p than CD63) and corresponding morphology. Platelet activation markers, including CD62p and CD63, may be localised to different regions within a forming thrombus. The inner core, subject to aggregation using selectins, would therefore present with a low level of detectable CD62p, while the outer shell would potentially express higher levels of CD62p and CD63 for further activation, the recruitment of platelets, and subsequent aggregation [66]. The apparent loss in CD62p (as suggested by a lower IPA) may be a reflection of its role in adhesion, and this was further indicated by fully spread hyalomeres in platelets with this treatment, as well as evidence of platelet aggregation [21,67].

The general higher levels of the CD63 IPA may have been due to various sources such as microparticles [68], which were also evident under scanning electron microscopy. In addition to mediating early and final events of platelet activation such as platelet recruitment and fibrinolysis [6,49], CD63 is associated with signalling in cancer [69]. Therefore, the higher levels of CD63, despite treatment, may suggest a failure to inhibit CD63-mediated signalling (which involves intracellular trafficking), thus mediating tumour survival and metastasis [69,70]. Thus far, a combined anastrozole, aspirin, and clopidogrel cocktail has been suggested to induce pro-coagulatory effects in the tumour microenvironment, contrary to the effects of aspirin and clopidogrel in cardiovascular disease alone [16,17,24]. Our results contrasted with studies that indicated that aspirin reduces thrombin-mediated platelet activation [63] and that clopidogrel reduces CD62p expression by inhibiting alpha granule exocytosis [71]; however, this has been proposed as a factor of combined therapies, notwithstanding the effects of the tumour cells themselves.

Within the hypercoagulatory environment, platelets are a source of various cytokines through the degranulation of their internal granules [51,54,72,73,74,75]. These cytokines play important roles in maintaining an inflammatory tumour microenvironment, modulating immune responses, and mediating tumour growth and survival [51,54,72,73,74,75]. An assessment of cytokine levels in whole blood exposed to anastrozole, aspirin, and clopidogrel-treated cells showed that IL-6-, PDGF-BB-, and TGFβ3-mediated responses in both cell lines, with T47D cells further inducing IL-4, TNF-α, VEGF-A, and TGF-α. This finding suggests that in addition to hypercoagulation, combined hormone and antiplatelet therapy may not prevent tumour progression. Furthermore, AnaAspClop-treated MCF7 cells displayed evidence of a partial EMT, whereas AnaAspClop-treated T47D cells showed a trend towards the acquisition of mesenchymal markers (independent of TGFβ1) and could suggest a role for PAR1-mediated effects [34] and, furthermore, a more aggressive tumour profile in T47D cells [62].

These findings thus support studies that have shown that combined hormone and antiplatelet therapy may promote tumour survival [40,41,43,76,77]. While our results indicate the importance of multiple cytokines, these effects are also facilitated by TGFβ1, which acts as the main mediator of the EMT through Smad 2/3 signalling [21,78]. Cells may undergo a complete EMT, whereby they lose epithelial features and acquire mesenchymal features, or they may undergo a partial EMT, which is characterised by a partial loss and/or gain of epithelial and mesenchymal features [79,80,81]. A partial EMT has also been related to a collective migration strategy [81] and associated with more aggressive tumour phenotypes. AnaAspClop treatment in T47D cells was shown to promote ultrastructural features associated with collective migration, with atopaxar having a similar effect [77].

Atopaxar targets the most potent pathway of platelet activation by blocking PAR1 (the main mediator of thrombin induced platelet activation), thus inhibiting thrombin binding [82]. AnaAto treatment in MCF7 cells increased detectable thrombin levels in whole blood compared to controls. While this may have reflected the non-binding of thrombin [62], an increase in circulating levels is associated with higher risk for hypercoagulation [64]. This was reflected in the platelet ultrastructure, which showed platelets at various stages of activation (from early to advanced). Notably, the antiplatelet control (Ato), despite inhibiting thrombin receptor-activating peptides (TRAP) and thrombin-mediated platelet aggregation [83], did not entirely prevent clot formation and was further associated with fibrin formation mediated by MCF7 cells. This finding suggests that the inhibiting concentration of atopaxar [83] may be insufficient to block the totality of PAR1 receptors and further suggests possible effects of tumour cells in facilitating hypercoagulation. Thrombin, which mediates various processes associated with hypercoagulation and tumour progression [84,85,86], was lower in whole blood exposed to AnaAto-treated T47D cells, with hypercoagulation evidenced by clot formation through platelet aggregates. The reduced circulating thrombin in whole blood exposed to AnaAto-treated T47D cells may indicate thrombin binding to PAR-1 receptors on T47D cells in order to facilitate cancer signalling and promote survival [23,85,87]. Therefore PAR receptor agonists may be targeted as part of adjuvant therapy against tumours to prevent PAR receptor-mediated signalling [6,88,89].

An assessment of platelet activation under AnaAto treatment revealed a similar trend to that observed with the AnaAspClop treatment, in that the CD62p IPA levels were generally lower and the CD63 IPA was higher, with platelet morphology revealing aggregates, fully spread platelets, and abundant microparticles. While atopaxar exhibits beneficial effects against platelet aggregation in patients suffering with thromboembolic disorders [23,90,91], the findings of this study suggest that this beneficial effect may be attenuated in the cancer setting due to the cancer therapy or the tumour cells themselves. Furthermore, high levels of CD63, as found in this study, are associated with the mediation of tumour progression and the EMT, being elevated in cancer patients [18,69]. In this study, IL-6, TNFα, and TGFβ3, all implicated in the EMT, were main mediators of responses in both cell lines. In addition, PDGF-BB was further associated with driving change in MCF7 cells, whereas VEGF-A and TGF-α were associated with mediating responses in T47D cells. AnaAto treatment did not prevent the secretion of tumour promoting cytokines in both cell lines, although more cytokines influenced MCF7 cell responses compared to T47D cells. This suggests that this treatment may increase aggressiveness or tumour survival associated with angiogenesis and the induction of pathways involved in proliferation and differentiation [21,22,23,24,51,65,72,81]. This finding corresponded with an assessment of genes associated with the EMT that showed an induction of a partial EMT in MCF7 cells. While these genes were downregulated in T47D cells, the cytokine expression of proteins involved in tumour survival was evident. Moreover, atopaxar alone has been shown to change T47D morphology from epithelial-like to rounded cell clusters [77], which may reflect the induction of collective cell migration and heightened aggression [81].

It is noteworthy that with all the used treatments, the heterogeneous response between MCF7 and T47D cells was evident. Even though these cell lines broadly represent the luminal A subtype breast cancer, our result highlighted how they modulate different responses to antiplatelet treatments in conjunction with anastrozole, with MCF7 cells being more susceptible to the aspirin and clopidogrel cocktail and T47D cells being more susceptible to atopaxar treatment. These findings highlight differences in the expression of potential drug targets [92] and indicate that the further consideration of this cancer subtype needs to be made in terms of clinical management. Moreover, these results highlight that the utility of antiplatelet therapy in cardiovascular disease cannot be applied to cancer management without the further consideration of drug interactions and the effects of the tumour cells themselves.

## 4. Materials and Methods

### 4.1. Patient Recruitment and Demographics

Ethical clearance was obtained from the Human Research Ethics Committee, University of the Witwatersrand, clearance number: M150263. Six healthy female participants (19–30 years of age) were recruited from the Faculty of Health Sciences, University of the Witwatersrand and signed a consent form prior to participating in this study. Exclusion criteria included pregnancy, smoking, use of oral contraceptives or injection, immunodeficiency, autoimmune disease, cancer or previous history of cancer, and consumption of anti-platelet and anti-coagulation medication (e.g., aspirin and warfarin).

### 4.2. Blood Collection

Peripheral blood was collected from participants between days 1 and 10 of their menstrual cycle, i.e., during the follicular phase whereby oestrogen and progesterone levels are at the lowest and platelets have less affinity for fibrinogen [93]. Peripheral blood was collected by venepuncture into 3.2% sodium citrate vacutainers (4.5 mL) (Biocom Africa, Centurion, South Africa, AUGP17002) using a 20-gauge needle by a phlebotomist at the Day Ward, Charlotte Maxeke Johannesburg Academic Hospital. The first 2 mL of blood were discarded to avoid the inclusion of mechanically activated platelets, and three vials of blood were then collected.

### 4.3. Experimentation

The MCF7 (P37) and T47D (P45) breast cancer cell lines were purchased from the American Type Culture Collection (ATCC). MCF7 cells were cultured in Dulbecco’s Medium Essential Media (DMEM) (Lonza, Kempton Park, South Africa, BE12-604F) supplemented with 10% foetal bovine serum (FBS) (Thermo Fisher Scientific, Fairland, South Africa, 10499044) and 0.1% penicillin/streptomycin (P/S) (Lonza, OMB 206). T47D cells were cultured in Roswell Park Memorial Institute media (RPMI) (Lonza, BE12-115F) supplemented with 10% FBS (Thermo Fisher Scientific, 10499044), 0.1% P/S (Lonza, OMB 206), and 0.2 U/mL human insulin (Novo Nordisk, Sanofi-Aventis, Midrand, South Africa).

For experimentation, MCF7 and T47D cells were seeded at 1 × 10^5^/well in a 24-well plate. Cells were treated with Cmax concentrations of 16.16 ng/mL anastrozole [77] (Sigma-Aldrich, Kempton Park, South Africa, A2736), 4.4µg/mL aspirin [94] (Sigma-Aldrich, A2093), 1.86ng/mL clopidogrel bisulphate [95] (Sigma-Aldrich, PHR1431), and 0.064 µM atopaxar hydrobromide [89] (Axon Medchem, Groningen, The Netherlands, 2030) for 24 h. Controls included a culture-media-only control and a diluent control corresponding to the final percentage of DMSO (0.03% DMSO) in working solutions. For experimentation, 300 µL whole blood (WB) were exposed to treated breast cancer cells for 5 min at room temperature (20–25 °C) [60,67]. WB was subsequently retained for analyses.

### 4.4. Thrombin Activity Assay

Whole blood was snap-frozen in liquid nitrogen following experimentation and stored at −80 °C. For the assay, samples were thawed at room temperature. Thrombin activity was measured using a fluorometric thrombin activity assay kit (Abcam, Centurion, South Africa ab197006), and the manufacturer’s protocol was followed. Briefly, standards were prepared, and reaction wells were prepared by mixing 5 µL of whole blood samples with 45 µL of a thrombin assay buffer (1:10 dilution). A master mix containing 5 µL of the thrombin substrate and 45 µL of the thrombin assay buffer was prepared and added into each well. Fluorescence was measured in a Fortega Glomax Explorer fluorescence spectrophotometer in the kinetic mode at an excitation wavelength of 350 nm and an emission wavelength of 450 nm. Readings were obtained at regular intervals for duration of 60 min at 37 °C. Data were managed in Microsoft Excel 2010 and analysed as specified by the manufacturer. The mean absorbance value of the blank was subtracted from all values to create the corrected absorbance for each sample.

### 4.5. Platelet Ultrastructure

For morphological assessment, 20 µL of lysed whole blood samples were pipetted onto glass coverslips into 24-well plates and incubated at 37 °C for 5 min for adhesion. Coverslips were washed in 0.1 M PBS by agitation on a micro-plate shaker at 1000 rpm for 20 min to remove trapped proteins and debris. Samples were fixed in a 1:1 ratio of 2.5% formaldehyde/glutaraldehyde (FA/Glut) for 15 min at room temperature and then rinsed three times in 1 M PBS at pH 7.2. Samples were fixed in 1% osmium tetroxide for 15 min and rinsed three times in distilled water. Subsequently, samples were dehydrated in graded alcohol series (30%, 50%, 70%, 80% 90%, and 95%) for 3 min each, with three changes in 100% ethanol for 5 mins each, and dried in hexamethyldisilazane (HMDS) for 1 h. Coverslips were mounted onto aluminium stubs using carbon tape and coated with a double layer of carbon using a Quorum sputter coater. Platelet ultrastructure was imaged using a Zeiss Gemini Ultra Plus Scanning Electron Microscope (Laboratory for Microscopy and Microanalysis, University of Pretoria), and images were taken at 1 KV.

### 4.6. Flow Cytometry

For the analysis of platelet activation from whole blood samples, erythrocytes were lysed using 3 mL of an ammonium chloride lysis buffer (1:10) for 10 min, and samples were centrifuged for 5 min at 200× *g*. The supernatant was discarded, and the pellet resuspended in 150 µL of Tyrode’s buffer. Positive control and technical control samples were prepared by incubating 0.1 U/mL human thrombin with lysed whole blood resuspended in 150 µL of Tyrode’s buffer for 5 min at room temperature to induce platelet activation [66].

Thereafter, 180 µL of lysed whole blood was incubated with optimal volumes of monoclonal antibodies (determined by titration): 10 µL of APC-mouse anti-human CD41a (BD Biosciences, Woodmead, South Africa, 559777), 10 µL of FITC-mouse anti-human CD62p (BD Biosciences, 555523), and 2.5 µL of PE-CY7 mouse anti-human CD63 (BD Biosciences, 561982) for 15 min at room temperature. Thereafter, samples were fixed in 0.5% paraformaldehyde for 10 min and centrifuged at 200× *g* for 5 min, and the pellet was resuspended in 500 µL of Tyrode’s buffer. Samples were stored at 4 °C overnight for analysis the following day on an LSR Fortessa flow cytometer (Department of Surgery, University of the Witwatersrand) equipped with four lasers (blue, red, violet, and UV).

Tracking beads diluted in sheath fluid were run prior to each experiment to check the status of the detectors (cytometer settings: FSC = 300 v; SSC = 275 v; APC = 460 v; FITC = 469 v; PECY7 = 674 v) and ensure optimal function. FACS Diva software was used to capture results and compensation controls (single stained technical controls; CD41a-APC only/CD62p-FITC only/CD63-PECY7 only and unstained) were used to correct for spectral overlap and determine optimal voltages. The following voltages were used to obtain data: FSC (approximate measure of cell size) 300 V, SSC (approximate measure of internal complexity/granularity) 275 V, APC 460 V, FITC 469 V, and PECY7 674 V. A total of 1 × 10^5^ events were measured for each sample. The threshold was set at 500.

**Figure 8:**
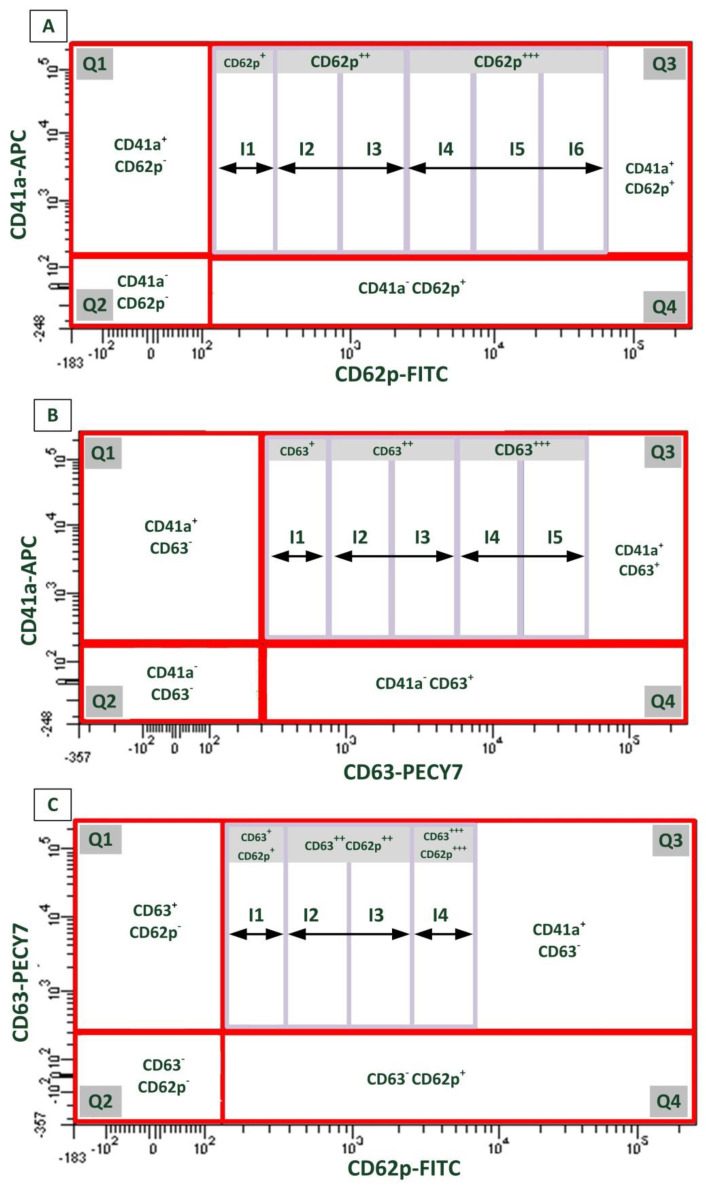
Scatterplots showing gating strategy used to investigate changes in expression levels of activation markers: CD62p and CD63 in platelets (CD41a). (**A**) Representative quadrants and intervals used to show the expression of CD62p only in CD41a platelets; (**B**) representative quadrants and intervals used to show the expression of CD63 only in CD41a platelets; and (**C**) representative gates and intervals used to define CD41a platelets expressing both CD62p and CD63.

The employed gating strategy was as follows: putative platelets were gated by drawing a gate (P1) around a population presenting low FSC and SSC properties (Supplementary Data, Figure S1). A singlet population designated P2 was gated (Supplementary Data, Figure S1), based on P1, in order to minimise data loss due to cell clumping. The independent expression of either CD62p or CD63 was represented in scatterplots based on the singlet population (P2). These scatter-plots showed platelets (CD41a^+^) that were expressing either CD62p or CD63 (designated Q3 in Figure 8). The graded expression levels of CD62p and/or CD63 were represented within intervals 1–4 (Figure 8). Intervals were determined based on the spread of each population within the quadrant [66].

Acquired data were exported to Microsoft Excel for data management. The IPA [96], which describes the geometric mean fluorescence intensity of activation marker expression in direct relation to the number of platelets expressing the marker, was calculated for each quadrant (Q3) and interval (I1-6) [66] as follows:

IPA CD62p = gMFI (CD62p)*n(CD41a^+^CD62^+^) and IPA (CD63) = gMFI (CD63)*n(CD41a^+^CD63^+^).

### 4.7. Data Analysis for Thrombin Concentration and Platelet Activation

Statistical analyses were performed using the PAST3 software. A Shapiro–Wilk test of normality determined that the data were not normally distributed. The non-parametric Friedman test and corresponding post hoc test were conducted in order to determine whether the index of platelet activation and thrombin activity were significantly altered following platelet exposure to hormone therapy and antiplatelet therapy-treated breast cancer cells. Significance was set at *p* < 0.05.

### 4.8. Cytokine Analysis

Whole blood, along with conditioned media from controls that had been stored at −80 °C was thawed at RT prior to being used to determine cytokine expression levels. The following panels of cytokines were used: IL1β, TNF-α, PDGF-AB (R&D Systems, Inc., Minneapolis, MN, cat no. LXSAHM-03, Lot: L127797), IFN-β1, IL6, PDGF-BB, VEGF-A, IL4, IL10, TGF-α (R&D Systems, Inc., Minneapolis, MN, cat co. LXSAHM-07, Lot: L126911), and TGFβ -1, -2, and -3 (Bio-Plex Pro ™ TGF-β Panel 3-Plex, Bio-Rad Laboratories, Inc., Hercules, CA, cat no. 171W40001M). Manufacturer’s guidelines, as indicated in the product sheet, were followed in quantifying cytokine levels.

### 4.9. Data Analysis

Concentration data (ρg/mL) were censored by removing points regarded as out of range (OOR) being either below the lower limit of quantification or above the upper limit of quantification. For missing data values, mean value imputation was employed only where ≥50% data points were present in any one culture group. This resulted in the removal of IL-1β from the analysis due to a number of non-detections. The dataset was then log transformed (log10) prior to performing a PCA using PAST v3.04 to explore the dataset [97]. The data were bootstrapped to an iteration of 1000, and PCA was conducted on a variance–covariance matrix whereby the dimensionality of the dataset was reduced generating PCs that could explain the majority of the variance in the dataset [98,99,100]. Scree plots were used to determine the number of PCs to retain. The contribution of each cytokine to these newly derived components was assessed using factor loadings. To determine the appropriate cut-off level for the assessment of cytokine contribution to the derived components, the following equation was used: $\mathrm{Cut}-\mathrm{off}\;\mathrm{level}=\frac{1}{\sqrt{n\left(\mathrm{cytokines}\right)}}-1$.

### 4.10. Gene Expression Using qPCR

Following cell treatment with hormone and antiplatelet drugs for 24 h, samples were incubated with whole blood for 5 min. Cells were subsequently rinsed in PBS (pH 7.4) thrice, then lysed with 350 µL of an RLT buffer (containing beta-mercaptoethanol) using a 22 gauge needle. The lysed cell homogenate was stored at −80 °C until further RNA extraction (~6–7 months). RNA was extracted from ~150,000 cells per treatment group using the Qiagen RNeasy kit (Qiagen, cat no. 74104), and the manufacturer’s guidelines were followed. Samples in triplicate were pooled for each treatment group (starting cell number >150,000). Complementary deoxyribonucleic acid (cDNA) was prepared using the High Capacity Reverse Transcription Kit (4368814, Applied Biosystems) according to the manufacturer’s guidelines. Primers were designed on the Eurofins website (www.eurofinsgenomics.com, accessed on 28 February 2021) and then validated using the oligoanalyzer tool on the IDT website (www.idtdna.com, accessed on 28 February 2021). A primer blast was conducted on PubMed (www.ncbi.nlm.nih.gov, accessed on 28 February 2021) for further validation and authentication of the primers (Table 7).

The PCR reaction was prepared in duplicate for six volunteers to a final volume of 10 µL (SPL096150, Thermo Fischer Scientific) in each well within a Piko plate (Thermo Fisher, Fairland, South Africa, cat no.SPL096150). Each reaction contained RNase-free water, 10 µM forward primer, 10 µM reverse primer, 2 ng of cDNA, and 2X SYBR green (A25742, applied Biosystems). The qPCR reaction was conducted for 40 cycles in the (PikoReal Real-Time PCR System, Thermo Fisher).

### 4.11. Data Analysis

The fold change in gene expression was calculated as outlined in [101]. The fold change represents the expression ratio of the gene of interest in relation to the untreated control. Thereafter, the mean of the fold change was calculated and missing data or outliers were imputed, provided that missing values were not 50% or more of the whole dataset [102]. The data were subsequently analysed in the PAST3 statistical software using the Friedman test and corresponding post hoc test (Wilcoxon signed rank test) in order to determine whether mRNA levels for E-cadherin, N-cadherin, vimentin, and TGFβ1 were altered by in breast cancer cells in response to treatment with anastrozole combined with antiplatelet therapy (aspirin and clopidogrel/atopaxar).

## Figures and Tables

**Table 1:** Index of platelet activation (IPA) indicated by CD62p in treatment groups and controls. The total CD62p IPA and within intervals 1–4. Data are represented as IPA ± standard deviation (SD).

	Total IPA (Q3) CD41^+^CD62p^+^		Interval 1 IPA (CD41a^+^CD62p^+^)		Interval 2 IPA (CD41a^++^CD62p^++^)		Interval 3 IPA (CD41a^++^CD62p^++^)		Interval 4 IPA (CD41a^+++^CD62p^+++^)	
	Whole Blood									
Untreated	1.45 × 10^6^ ± 2.88 × 10^5^		4.43 × 10^5^ ± 7.95 × 10^4^		7.57 × 10^5^ ± 6.60 × 10^4^		7.47 × 10^5^ ± 2.50 × 10^5^		1.76 × 10^4^ ± 1.06 × 10^4^	
Thrombin	1.83 × 10^6^ ± 1.06 × 10^6^		1.42 × 10^5^ ± 4.30 × 10^4^		6.20 × 10^5^ ± 8.51 × 10^4^		9.42 × 10^5^ ± 3.61 × 10^5^		6.03 × 10^5^ ± 2.90 × 10^5^	
	**Whole Blood Exposed to Breast Cancer Cells**									
	**MCF7**	T47D	**MCF7**	**T47D**	**MCF7**	**T47D**	**MCF7**	**T47D**	**MCF7**	**T47D**
Media	1.68 × 10^6^ ± 5.77 × 10^5^	1.70 × 10^6^ ± 9.03 × 10^5^	6.38 × 10^5^ ± 2.08 × 10^5^	5.77 × 10^5^ ± 1.93 × 10^5^	1.07 × 0^6^ ± 1.73 × 10^5^	6.69 × 10^5^ ± 3.22 × 10^5^	7.42 × 10^5^ ± 9.52 × 10^4^	2.87 × 10^5^ ± 1.13 × 10^5^	1.23 × 10^5^ ± 4.56 × 10^4^	2.61 × 10^4^ ± 2.71 × 10^4^
Diluent	1.63 × 10^6^ ± 3.91 × 10^5^	1.20 × 10^6^ ± 4.19 × 10^5^	5.79 × 10^5^ ± 1.39 × 10^5^	4.90 × 10^5^ ± 1.39 × 10^5^	8.44 × 10^5^ ± 2.43 × 10^5^	8.41 × 10^5^ ± 2.21 × 10^5^	4.10 × 10^5^ ± 1.19 × 10^5^	3.27 × 10^5^ ± 1.11 × 10^5^	3.22 × 10^4^ ± 3.74 × 10^4^	7.52 × 10^4^ ± 4.45 × 10^4^
Ana	1.72 × 10^6^ ± 2.63 × 10^5^	1.71 × 10^6^ ± 4.14 × 10^5^	5.75 × 10^5^ ± 1.77 × 10^5^	5.73 × 10^5^ ± 1.59 × 10^5^	8.10 × 10^5^ ± 7.44 × 10^4^	8.08 × 10^5^ ± 1.55 × 10^5^	5.22 × 10^5^ ± 1.64 × 10^5^	4.48 × 10^5^ ± 2.64 × 10^5^	8.14 × 10^4^ ± 4.22 × 10^4^	1.28 × 10^5^ ± 5.83 × 10^4^
AnaAspClop	1.66 × 10^6^ ± 3.23 × 10^5^	1.36 × 10^6^ ± 4.35 × 10^5^	5.17 × 10^5^ ± 2.01 × 10^5^	4.62 × 10^5^ ± 1.31 × 10^5^	7.80 × 10^5^ ± 1.35 × 10^5 [~]^	7.75 × 10^5^ ± 1.02 × 10^5^	3.39 × 10^5^ ± 1.30 × 10^5 [# ~]^	4.92 × 10^5^ ± 2.14 × 10^5^	2.23 × 10^5^ ± 1.45 × 10^5 [# $ ^]^	2.59 × 10^4^ ± 2.15 × 10^4 [*]^
AspClop	1.47 × 10^6^ ± 2.75 × 10^5^	1.30 × 10^6^ ± 5.20 × 10^5^	3.99 × 10^5^ ± 1.50 × 10^5^	4.34 × 10^5^ ± 1.14 × 10^5^	7.02 × 10^5^ ± 2.39 × 10^5^	7.73 × 10^5^ ± 1.73 × 10^5^	5.05 × 10^5^ ± 1.80 × 10^5^	3.39 × 10^5^ ± 1.32 × 10^5^	2.17 × 10^4^ ± 2.06 × 10^4^	1.74 × 10^4^ ± 1.11 × 10^4^
AnaAto	1.73 × 10^6^ ± 8.26 × 10^5^	1.46 × 10^6^ ± 5.65 × 10^5^	4.53 × 10^5^ ± 9.15 × 10^4^	5.16 × 10^5^ ± 1.55 × 10^5 [^]^	8.39 × 10^5^ ± 1.37 × 10^5^	8.15 × 10^5^ ± 2.29x10^5^	4.31 × 10^5^ ± 1.05 × 10^5 [# ~]^	4.08 × 10^5^ ± 1.63 × 10^5 [# ~]^	1.20 × 10^4^ ± 1.20 × 10^4^ [~]	1.18 × 10^4^ ± 5.07 × 10^3 [*]^
Ato	1.37 × 10^6^ ± 3.67 × 10^5^	1.42 × 10^6^ ± 5.62 × 10^5^	4.19 × 10^5^ ± 1.03 × 10^5^	4.71 × 10^5^ ± 9.30 × 10^4^	6.92 × 10^5^ ± 1.24 × 10^5^	9.03 × 10^5^ ± 1.65 × 10^5^	7.75 × 10^5^ ± 2.06 × 10^5^	8.07 × 10^5^ ± 1.31 × 10^5^	6.31 × 10^4^ ± 4.21 × 10^4^	2.03 × 10^4^ ± 1.28 × 10^4^

Annotations: +/++/+++ refers to levels of markers, + = positive, ++ = medium expression, +++ high expression. $ = significantly different compared to untreated whole blood (*p* < 0.05); ^ = significantly different compared to diluent control (DMSO) (*p* < 0.05); ~ = significantly different compared to untreated cells (MCF7/T47D) (*p* < 0.05); * = significantly different compared to hormone therapy control (*p* < 0.05); **#** = significantly different compared to antiplatelet therapy control (*p* < 0.05); red = cell lines significantly different at matched treatments (*p* < 0.05).

**Table 2:** Index of platelet activation (IPA) indicated by CD63 in treatment groups and controls. The total CD63 IPA and within intervals 1–4. Data are represented as IPA ± standard deviation (SD).

	Total IPA CD41^+^CD63^+^ (Q3)		Interval 1 IPA (CD41a^+^CD63^+^)		Interval 2 IPA (CD41a^++^CD63^++^)		Interval 3 IPA (CD41a^++^CD63^++^)		Interval 4 IPA (CD41a^+++^CD63^+++^)	
	Whole Blood									
Untreated	2.77 × 10^6^ ± 1.11 × 10^6^		6.13 × 10^5^ ± 1.19 × 10^5^		1.31 × 10^6^ ± 2.19 × 10^5^		1.49 × 10^6^ ± 7.37 × 10^5^		1.33 × 10^6^ ± 3.75 × 10^5^	
Thrombin	7.54 × 10^6^ ± 3.91 × 10^6^		2.13 × 10^5^ ± 7.41 × 10^4^		7.45 × 10^5^ ± 1.49 × 10^5^		2.42 × 10^6^ ± 7.34 × 10^4^		3.50 × 10^6^ ± 1.34 × 10^6^	
	**Whole Blood Exposed to Breast Cancer Cell Lines**									
	**MCF7**	**T47D**	**MCF7**	**T47D**	**MCF7**	**T47D**	**MCF7**	**T47D**	**MCF7**	**T47D**
Media	3.05 × 10^6^ ± 1.22 × 10^6^	4.46 × 10^6^ ± 2.67 × 10^6^	9.38 × 10^5^ ± 1.42 × 10^5^	6.00 × 10^5^ ± 1.39 × 10^5^	1.62 × 10^6^ ± 5.77 × 10^5^	1.01 × 10^5^ ± 5.61 × 10^5^	1.25 × 10^6^ ± 5.14 × 10^5^	1.24 × 10^6^ ± 5.68 × 10^5^	1.05 × 10^6^ ± 2.81 × 10^5^	9.67 × 10^5^ ± 2.33 × 10^5^
Diluent	3.87 × 10^6^ ± 2.12 × 10^6^	3.77 × 10^6^ ± 1.17 × 10^6^	6.51 × 10^5^ ± 1.03 × 10^5^	6.37 × 10^5^ ± 1.93 × 10^5^	1.77 × 10^6^ ± 5.19 × 10^5^	1.30 × 10^6^ ± 3.23 × 10^5^	1.78 × 10^6^ ± 4.75 × 10^5^	1.65 × 10^6^ ± 6.43 × 10^5^	1.29 × 10^6^ ± 4.74 × 10^5^	1.10 × 10^6^ ± 2.74 × 10^5^
Ana	4.72 × 10^6^ ± 9.84 × 10^5^	3.67 × 10^6^ ± 1.65 × 10^6^	6.59 × 10^5^ ± 1.17 × 10^5^	6.41 × 10^5^ ± 1.89 × 10^5^	1.65 × 10^6^ ± 3.14 × 10^5^	1.41 × 10^6^ ± 4.06 × 10^5^	1.96 × 10^6^ ± 8.20 × 10^5^	1.65 × 10^6^ ± 6.30 × 10^5^	1.50 × 10^6^ ± 6.07 × 10^5^	1.38 × 10^6^ ± 3.81 × 10^5^
AnaAspClop	4.97 × 10^6^ ± 2.17 × 10^6^	3.86 × 10^6^ ± 1.34 × 10^6^	8.35 × 10^5^ ± 2.15 × 10^5^	6.22 × 10^5^ ± 2.18 × 10^5^	1.36 × 10^6^ ± 2.12 × 10^5 [^]^	1.41 × 10^6^ ± 3.66 × 10^5^	2.02 × 10^6^ ± 6.83 × 10^5^	1.73 × 10^6^ ± 2.52 × 10^5^	1.47 × 10^6^ ± 4.24 × 10^5^	1.47 × 10^6^ ± 3.59 × 10^5 [~]^
AspClop	3.62 × 10^6^ ± 2.11 × 10^6^	3.54 × 10^6^ ± 1.44 × 10^6^	4.93 × 10^5^ ± 1.86 × 10^5^	5.57 × 10^5^ ± 1.63 × 10^5^	1.29 × 10^6^ ± 5.46 × 10^5^	1.39 × 10^6^ ± 4.00 × 10^5^	1.79 × 10^6^ ± 6.62 × 10^5^	1.83 × 10^6^ ± 5.63 × 10^5^	1.32 × 10^6^ ± 6.61 × 10^5^	1.22 × 10^6^ ± 3.58 × 10^5^
AnaAto	4.09 × 10^6^ ± 1.98 × 10^6^	3.92 × 10^6^ ± 1.67 × 10^6^	5.54 × 10^5^ ± 6.41 × 10^4 [~]^	6.19 × 10^5^ ± 1.73 × 10^5^	1.51 × 10^6^ ± 2.71 × 10^5^	1.47 × 10^6^ ± 4.13 × 10^5^	1.80 × 10^6^ ± 5.99 × 10^5^	1.90 × 10^6^ ± 7.20 × 10^5 [* ~]^	**2.01 × 10^6^ ± 6.70 × 10^5 [~]^**	**1.24 × 10^6^ ± 3.39 × 10^5^**
Ato	4.02 × 10^6^ ± 2.29 × 10^6^	4.36 × 10^6^ ± 2.43 × 10^6^	5.82 × 10^5^ ± 1.29 × 10^5^	5.59 × 10^5^ ± 1.95 × 10^5^	1.36 × 10^6^ ± 3.71 × 10^5^	1.52 × 10^6^ ± 3.91 × 10^5^	1.87 × 10^6^ ± 4.01 × 10^5^	2.00 × 10^6^ ± 5.07 × 10^5^	1.31 × 10^6^ ± 2.68 × 10^5^	1.56 × 10^6^ ± 5.08 × 10^5^

Annotations: +/++/+++ refers to levels of markers, being + = positive, ++ = medium expression, +++ high expression. $ = significantly different compared to untreated whole blood (*p* < 0.05); ^ = significantly different compared to diluent control (DMSO) (*p* < 0.05); ~ = significantly different compared to untreated cells (MCF7/T47D) (*p* < 0.05); * = significantly different compared to hormone therapy control (*p* < 0.05); **#** = significantly different compared to antiplatelet therapy control (*p* < 0.05); red = cell lines significantly different at matched treatments (*p* < 0.05).

**Table 3:** The relationship between the CD62p and CD63 IPA represented by the Spearman correlation value (r) and corresponding regression analysis (r^2^). A strong linear relationship was observed between the CD62p and CD63 IPA at late intervals (whereby platelets were highly activated), and there was a strong regression suggestion that the markers were dependent at these intervals.

	Interval 3 (CD41a^++^CD62p^++^CD63^++^)								Interval 4 (CD41a^+++^CD62p^+++^CD63^+++^)							
	(r)		*p*-Value		r^2^		% r^2^		(r)		*p*-Value		r^2^		% r^2^	
UT WB	0.96		0.0018		0.93		93.06		0.98		0.0006		0.96		96	
	**Whole Blood Exposed to Breast Cancer Cell Lines**															
	MCF7	T47D	MCF7	T47D	MCF7	T47D	MCF7	T47D	MCF7	T47D	MCF7	T47D	MCF7	T47D	MCF7	T47D
Media	0.79	0.95	0.06	0.00	0.63	0.91	63	91	0.76	1.00	0.08	0.00	0.58	0.99	58	99
Diluent	0.77	0.93	0.07	0.00	0.60	0.87	60	87	0.95	0.97	0.00	0.00	0.90	0.94	90	94
Ana	0.92	0.91	0.01	0.01	0.84	0.84	84	84	0.81	0.71	0.05	0.11	0.66	0.50	66	50
AnaAspClop	0.83	0.67	0.04	0.15	0.68	0.45	68	45	0.85	0.77	0.03	0.07	0.71	0.60	71	60
AspClop	0.71	0.85	0.11	0.03	0.51	0.73	51	73	0.99	0.88	0.00	0.02	0.98	0.78	98	78
AnaAto	0.91	0.65	0.01	0.16	0.84	0.42	84	43	0.98	0.90	0.00	0.01	0.97	0.81	97	81
Ato	0.25	0.81	0.62	0.05	0.06	0.65	72	66	0.93	0.85	0.00	0.03	0.86	0.72	86	72

Annotations: +/++/+++ refers to levels of markers, being + = positive, ++ = medium expression, +++ high expression. r-values between 0 and 1 represent the strength of the linear relationship, whereas r^2^ values represent the variance between the CD62p and CD63 IPA; 0–0.5 = weak; 0.51–0.79 = moderate; 0.8–1 = strong; bold = *p* < 0.05.

**Table 4:** Loadings from principal component (PC) analysis of cytokines of all cytokines from MCF-7 and T47D culture groups under anastrozole, aspirin, and clopidogrel treatment. Bold indicates contributing cytokines above the cut-off level. Underline indicates cytokines that contributed just below the cut-off level.

MCF-7				T47D			
Cytokines	PC1	PC2	PC3	Cytokines	PC1	PC2	PC3
**IL-6**	**0.57829**	−0.28231	**0.55872**	**IL-6**	**0.5052**	−0.01729	−0.24827
**IL-4**	0.089899	0.13978	−0.068816	**IL-4**	−0.12102	−0.11954	**0.38592**
**TNF-α**	−0.056041	0.301	−0.40729	**TNF-α**	−0.29173	−0.14179	**0.48693**
**VEGF-A**	−0.29084	0.12259	−0.2804	**VEGF-A**	**−0.44029**	−0.032	0.19185
**PDGF-AB**	0.0046572	0.024835	−0.056169	**PDGF-AB**	0.046581	−0.01932	0.066934
**PDGF-BB**	−0.25008	**0.67916**	**0.61872**	**PDGF-BB**	−0.3234	**0.89851**	−0.11626
**TGF-α**	0.14638	0.15606	−0.16061	**TGF-α**	0.055891	−0.01114	**0.38145**
**TGF-β1**	0.31812	0.24762	0.033047	**TGF-β1**	0.30042	0.21368	0.19563
**TGF-β2**	0.10754	0.092757	0.0076364	**TGF-β2**	0.12873	0.077109	0.066222
**TGF-β3**	**0.61075**	**0.48886**	−0.16022	**TGF-β3**	**0.48421**	0.32383	**0.5562**
**% Variance** **Explained**	42.389	22.534	14.607	**% Variance** **Explained**	32.719	23.543	16.53
**Total % Variance Explained**			79.53%	**Total % Variance Explained**			72.79%

**Table 5:** Loadings from principal component (PC) analysis of cytokines of all cytokines from MCF-7 and T47D culture groups under anastrozole and atopaxar treatment. Bold indicates contributing cytokines above the cut-off level. Underline indicates cytokines that contributed just below the cut-off level.

MCF-7				T47D			
Cytokines	PC1	PC2	PC3	Cytokines	PC1	PC2	PC3
**IL-6**	**0.57829**	−0.10469	**−0.45249**	**IL-6**	**0.67371**	0.04743	−0.020518
**IL-4**	0.089899	0.072779	0.083299	**IL-4**	−0.15703	−0.27196	0.28599
**TNF-α**	−0.056041	0.14778	**0.64389**	**TNF-α**	−0.23865	−0.26364	**0.52246**
**VEGF-A**	−0.29084	0.091886	0.14266	**VEGF-A**	**−0.44568**	−0.065366	−0.069505
**PDGF-AB**	0.0046572	−0.01001	0.0599	**PDGF-AB**	0.028896	−0.038475	0.11293
**PDGF-BB**	−0.25008	**0.87168**	**−0.37212**	**PDGF-BB**	−0.24729	0.9048	0.22641
**TGF-α**	0.14638	0.14149	**0.37571**	**TGF-α**	0.054262	−0.042487	**0.64427**
**TGF-β1**	0.31812	0.24711	0.14391	**TGF-β1**	0.27747	0.15562	0.15452
**TGF-β2**	0.10754	0.073908	0.051364	**TGF-β2**	0.12104	0.052502	0.050477
**TGF-β3**	**0.61075**	0.32709	0.21633	**TGF-β3**	0.3306	0.033036	**0.36669**
**% Variance** **explained**	41.672	24.226	15.783	**% Variance** **explained**	38.341	26.436	*11*.855
**Total % Variance Explained**			81.68%	**Total % Variance Explained**			76.63%

**Table 6:** Fold change in the expression of genes involved in facilitating epithelial to mesenchymal transitions (TGFβ1, E-cadherin, N-cadherin, and vimentin) in breast cancer cells (MCF7 and T47D) treated with anastrozole and antiplatelet therapy drugs (aspirin and clopidogrel/atopaxar) and exposed to whole blood. Data are represented as log fold change ± standard deviation (SD).

	Log TGFβ1		Log E-cadherin		Log N-cadherin		Log Vimentin	
	MCF7	T47D	MCF7	T47D	MCF7	T47D	MCF7	T47D
Media	0.00 ± (−0.56)	0.01 ± (−0.66)	0.08 ± (−0.29)	0.09 ± (−0.44)	0.45 ± 0.26	0.38 ± 0.14	0.16 ± 0.15	0.34 ± 0.04
Diluent	−0.25 ± (−0.78)	−0.03 ± (−0.52)	0.02 ± (−0.70)	0.46 ± (−0.10)	−0.22 ± (−0.02)	−0.12 ± (−0.21)	−0.10 ± (−0.07)	0.31 ± 0.07
Ana	−0.10 ± (−0.55)	−0.03 ± (−0.76)	0.02 ± (−0.94)	0.08 ± (−0.45)	0.13 ± (−0.09)	0.43 ± 0.25	0.18 ± 0.27	0.32 ± (−0.02)
AnaAspClop	−0.15 ± (−0.37)	−0.13 ± (−0.87) ^[$, ~]^	0.13 ± (−0.15)	−0.11 ± (−0.74) ^[^]^	0.49 ± 0.65 ^[^]^	0.02 ± (−0.33)	0.25 ± 0.30	0.08 ± (−0.43) ^[#]^
AspClop	0.02 ± (−0.67)	−0.08 ± (−0.77)	0.11 ± (−0.19)	−0.06 ± (−0.76)	0.37 ± 0.39	−0.24 ± (−0.38)	−0.14 ± (−0.53)	−0.06 ± (−0.60)
AnaAto	−0.13 ± (−0.55)	−0.15 ± (−0.59) ^[^]^	0.16 ± (−0.01)	−0.19 ± (−0.76) ^[^, ~]^	0.02 ± (−0.39)	−0.26 ± (−1.30) ^[~]^	0.19 ± 0.07	−0.17 ± (−0.74) ^[~, ^]^
Ato	0.02 ± (−0.32)	−0.10 ± (−0.64)	0.25 ± (−0.04)	−0.08 ± (−0.56)	0.50 ± 0.47	−0.23 ± (−0.44)	0.21 ± 0.08	0.00 ± (−0.22)

Annotations: Highlighted blocks show downregulated genes; $ = significantly different compared to untreated whole blood (*p* < 0.05); ^ = significantly different compared to diluent control (DMSO) (*p* < 0.05); ~ = significantly different compared to untreated cells (MCF7/T47D) (*p* < 0.05); * = significantly different compared to hormone therapy control (*p* < 0.05); **#** = significantly different compared to antiplatelet therapy control (*p* < 0.05); red = cell lines significantly different at matched treatments (*p* < 0.05).

**Table 7:** Primer sequences used for quantitative real-time polymerase chain reaction (qPCR).

Gene	Primer Sequence (5′-3′)	Accession Number
*ACTB* (Reference gene)	**F:** GGC CGA GGA CTT TGA TTG CAC **R:** TTA GGA TGG CAA GGG ACT TCC TGT	NM_001101.3
*RPLO* (Reference gene)	**F:** TGC AGC TGA TCA AGA CTG GAG ACA **R:** TCC AGG AAG CGA GAA TGC AGA GTT	BC001834.2
*GAPDH* (Reference gene)	**F:** TGC ACC ACC AAC TGC TTA GC **R:** GGC ATG GAC TGT GGT CAT GAG	NM_002046.5
TGFβ-1	**F:** 5′-CTCGCCAGAGTGGTTATCTT-3′ **R:** 5′-AGTGTGTTATCCCTGCTGTC-3′	NM_000660.7
Vimentin	**F:** 5′-CCT CTT CCA AAC TTT TCC TCC-3′ **R:** 5′-CGT TGA TAA CCT GTC CAT CTC-3′	NM_003380.5
E-cadherin	**F:** 5′-TTC CTC CCA ATA CAT CTC CC-3′ **R:** 5′-TTG ATT TTG TAG TCA CCC ACC-3′	NM_004360.5
N-cadherin	**F:** 5′-CAT CAT TGC CAT CCT GCT C-3′ **R:** 5′-TCT TCT TCT CCT CCA CCT TC-3′	XM_017025514.2

## Data Availability

The authors will make the data available, subject to approval from the Human Ethics Research Committee at the University of the Witwatersrand, should the data be required.

## Supplementary Materials

The following are available online at https://www.mdpi.com/article/10.3390/ijms22084153/s1, Figure S1: Representative scatter plots and histogram depicting the spread of whole blood.
