# Supplementary material for: Antiplatelet Therapy Combined with Anastrozole Induces Features of Partial EMT in Breast Cancer Cells and Fails to Mitigate Breast-Cancer Induced Hypercoagulation
Source: Int J Mol Sci. 2021 Apr 16;22(8):4153. doi: 10.3390/ijms22084153 (PMC8074114; doi:10.3390/ijms22084153)
Supplement: Supplementary file 1 [file ijms-22-04153-s001.zip › ijms-1146221-supplementary.pdf]

Supplementary figure

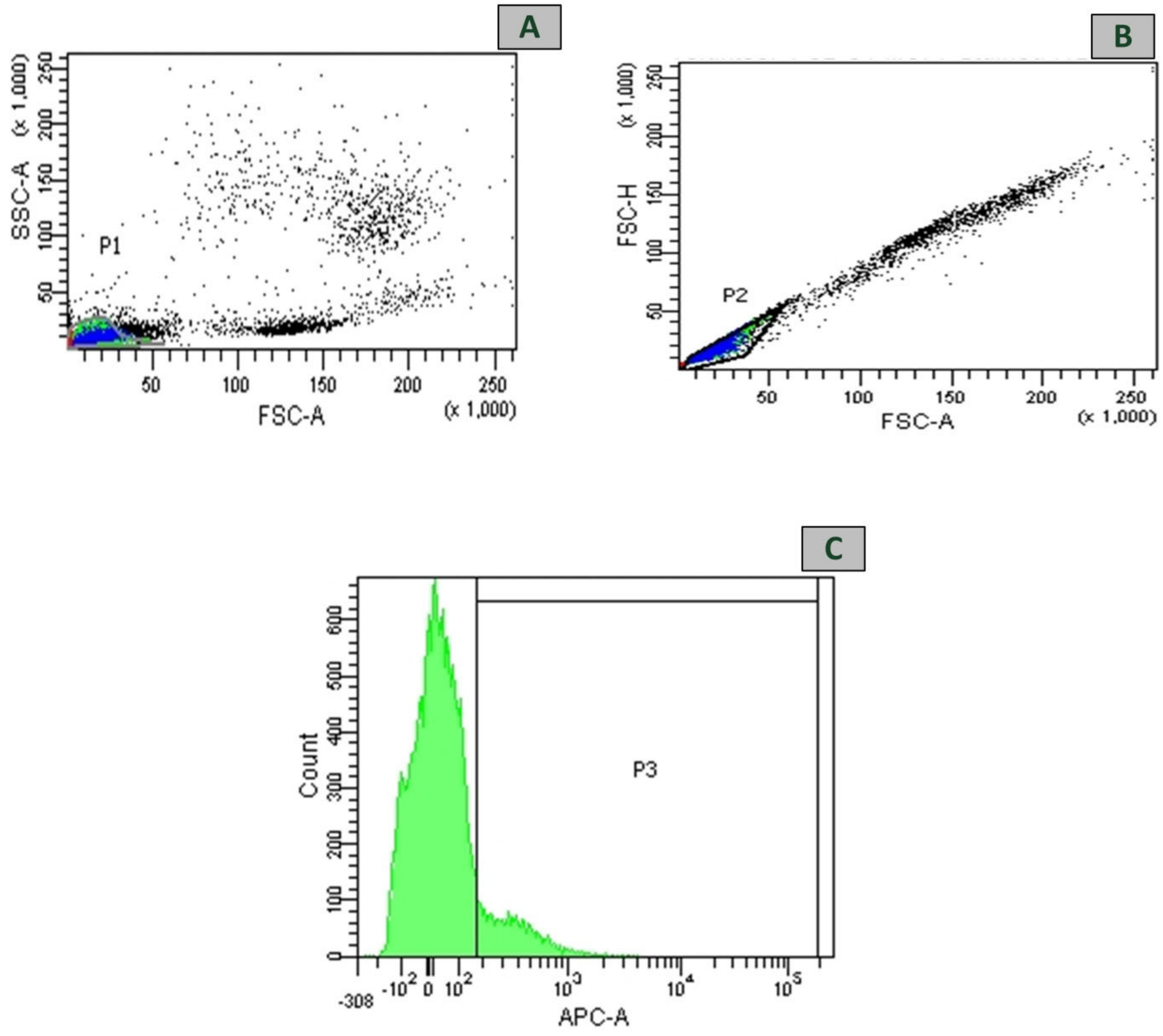

Figure S1. Representative scatter plots and histogram depicting the spread of whole blood. A) Platelets were gated on forward (FSC-A) and side scatter (SSC-A) properties. Due to their small size and less internal complexity platelets aggregate at the bottom left corner in the scatter plot (P1). **B)** The singlet population was gated (P2) using forward scatter area and height. Thus aggregated cells were excluded from the analysis in order to accurately analyse data. **C)** The histogram shows platelet population gated (P3) based on the expression of CD41a^{+} events.
